# Epidemiology and clinical presentation of the four human parainfluenza virus types

**DOI:** 10.1186/1471-2334-13-28

**Published:** 2013-01-23

**Authors:** Wen-Kuan Liu, Qian Liu, De-Hui Chen, Huan-Xi Liang, Xiao-Kai Chen, Wen-Bo Huang, Sheng Qin, Zi-Feng Yang, Rong Zhou

**Affiliations:** 1 State Key Laboratory of Respiratory Diseases (Guangzhou Medical University), 1 Kang Da Road, Guangzhou, Guangdong, 510230, China; 2 The First Affiliated Hospital of Guangzhou Medical University, 151 Yan Jiang Road, Guangzhou, Guangdong, 510230, China

## Abstract

**Background:**

Human parainfluenza viruses (HPIVs) are important causes of upper respiratory tract illness (URTI) and lower respiratory tract illness (LRTI). To analyse epidemiologic and clinical characteristics of the four types of human parainfluenza viruses (HPIVs), patients with acute respiratory tract illness (ARTI) were studied in Guangzhou, southern China.

**Methods:**

Throat swabs (n=4755) were collected and tested from children and adults with ARTI over a 26-month period, and 4447 of 4755 (93.5%) patients’ clinical presentations were recorded for further analysis.

**Results:**

Of 4755 patients tested, 178 (3.7%) were positive for HPIV. Ninety-nine (2.1%) samples were positive for HPIV-3, 58 (1.2%) for HPIV-1, 19 (0.4%) for HPIV-2 and 8 (0.2%) for HPIV-4. 160/178 (88.9%) HPIV-positive samples were from paediatric patients younger than 5 years old, but no infant under one month of age was HPIV positive. Seasonal peaks of HPIV-3 and HPIV-1 occurred as autumn turned to winter and summer turned to autumn. HPIV-2 and HPIV-4 were detected less frequently, and their frequency of isolation increased when the frequency of HPIV-3 and HPIV-1 declined. HPIV infection led to a wide spectrum of symptoms, and more “hoarseness” (p=0.015), “abnormal pulmonary breathing sound” (p<0.001), “dyspnoea” (p<0.001), “pneumonia” (p=0.01), and “diarrhoea” (p<0.001) presented in HPIV-positive patients than HPIV-negative patients. 10/10 (100%) HPIV-positive adult patients (≥14 years old) presented with systemic influenza-like symptoms, while 90/164 (54.9%) HPIV-positive paediatric patients (<14 years old) presented with these symptoms (p=0.005). The only significant difference in clinical presentation between HPIV types was “Expectoration” (p<0.001). Co-infections were common, with 33.3%–63.2% of samples positive for the four HPIV types also testing positive for other respiratory pathogens. However, no significant differences were seen in clinical presentation between patients solely infected with HPIV and patients co-infected with HPIV and other respiratory pathogens.

**Conclusions:**

HPIV infection led to a wide spectrum of symptoms, and similar clinical manifestations were found in the patients with four different types of HPIVs. The study suggested pathogenic activity of HPIV in gastrointestinal illness. The clinical presentation of HPIV infection may differ by patient age.

## Background

Human parainfluenza viruses (HPIVs) are RNA viruses in the genus *Paramyxoviridae*. Four HPIV types have been identified [1,2]. HPIVs are important causes of upper respiratory tract illness (URTI) and lower respiratory tract illness (LRTI), especially in children [3,4]. An estimated five million LRTI occur each year in the United States in children under 5 years old, and HPIVs have been isolated in up to one third of these infections [5-7]. The HPIV-1, HPIV-2 and HPIV-3 are second only to respiratory syncytial virus (RSV) as a cause of hospitalizations (2%–17%) for acute respiratory infection among children aged younger than 5 years in the United States [1,8-10].

Compared with studies of HPIV infection in children, less is known about infections in adults. Most HPIV infections in adults cause mild upper respiratory tract symptoms, but the elderly or those with compromised immune systems are at increased risk for severe HPIV infection [1,3,11-13]. Compared with types 1−3, only a small number of reports have studied HPIV-4 [14-18], and the lack of epidemiologic data on HPIV-4 prevents a clear understanding of the full clinical pattern of HPIVs. In addition, any differences in the clinical presentation of the four HPIV types are still largely unknown.

The aims of this study were to explore the epidemiologic features and clinical manifestations of HPIVs and other common respiratory pathogens in children and adults with acute respiratory tract illness (ARTI) in Guangzhou, southern China, and to uncover clues that might help to establish clinical distinctions between different HPIV types.

## Methods

### Respiratory sample collection

Samples in this study were taken as part of standard care. The First Affiliated Hospital of Guangzhou Medical University Ethics Committee approved the experimental design and patient involvement in this study. Written informed consent was obtained from the patient for publication of this report and any accompanying images.

Throat swab samples were collected from patients with ARTI (presenting with at least two of the following symptoms: cough, pharyngeal discomfort, nasal obstruction, coryza, sneeze, dyspnoea) at three hospitals in Guangzhou, southern China between July 2009 and August 2011. The samples were refrigerated at 2 to 8°C and transported on ice to State Key Laboratory of Respiratory Diseases and analysed every working day or stored at −80°C before testing. Over 97 samples were collected and tested during each month in our study.

Clinical presentations were collected and categorized retrospectively into the following six groups from the patients’ medical records using designed presentation cards: URTI, LRTI, systemic influenza-like symptoms, gastrointestinal illness, neurologic symptom and others. Patients with nasal obstruction, coryza, sneeze, cough, pharyngeal discomfort, or hoarseness were categorized as having URTI. Patients with pneumonia, bronchopneumonia, increasing lung markings, dyspnoea, or abnormal pulmonary breath sound were categorized as having LRTI. Patients with high fever (≥38°C), chills, dizziness, headache, myalgia or debilitation were categorized as having systemic influenza-like symptoms. Patients with vomiting, poor appetite, or diarrhoea were categorized as having gastrointestinal illness. Patients with convulsion were categorized as having an neurologic symptom. Patients with other symptoms, including but not limited to rash, were classified as “others”. Some patients were assigned to multiple clinical presentation groups. Pneumonia and bronchopneumonia were diagnosed by chest radiography. Pneumonia was defined as an acute illness with radiographic pulmonary shadowing which was at least segmental or present in one lobe (excluding the bronchi); bronchopneumonia was defined as inflammation of the walls of the smaller bronchial tubes, with varying amounts of pulmonary consolidation due to spread of the inflammation into peribronchiolar alveoli and the alveolar ducts. Other clinical symptoms were identified by common medical examinations and clinical descriptions.

### Real-time polymerase chain reaction (PCR) for detection of HPIV and other common respiratory pathogens

DNA or RNA from respiratory samples was extracted using QIAamp DNA Mini Kit or QIAamp Viral RNA Mini Kit (Qiagen Co. Ltd., Shanghai, China) in accordance with the manufacturer’s protocols. The four types of HPIV were tested by TaqMan real-time PCR as previously reported [19], and 13 other common respiratory pathogens were also selected for TaqMan real-time PCR testing, including influenza A virus (infA), influenza B virus (infB), respiratory syncytial virus (RSV), adenovirus (ADV), enterovirus (EV), human metapneumovirus (HMPV), four strains of human coronavirus (HCoV-229E, OC43, NL63 and HKU1), human bocavirus (HBoV), *Mycoplasma pneumoniae* (MP), and *Chlamydophila pneumoniae* (CP) [19].

### Statistical analysis

For comparisons of categorical data, χ^2^ test and Fisher’s exact test were used where appropriate. All tests were two-tailed and p<0.05 was considered statistically significant.

## Results

### Detection of HPIVs from patients with ARTI

We tested 4755 samples for HPIV and 13 other respiratory pathogens between July 2009 and August 2011 in Guangzhou, southern China. The median age was 4.75 (interquartile range, 1.00 to 25.00), and ranged from one day to 91 years. Pathogens were detected in 2439/4755 (51.3%) samples, and were detected in a higher proportion of samples from children (<14 years old) (1503/2793; 53.8%) than from adults (≥14 years old) (936/1962; 47.7%) (p<0.001). The pathogens identified most frequently were infA (833/4755; 17.5%), RSV (524/4755; 11.0%) and MP (274/4755; 5.8%) (Table 1).

**Table 1:** Detection of respiratory pathogens by real-time PCR

**Pathogens**^**#**^	Number of positive samples with potential pathogens^*^																	**Detection rate (%)**
	**HPIV-1**	**HPIV-2**	**HPIV-3**	**HPIV-4**	**infA**	**infB**	**RSV**	**ADV**	**HMPV**	**HBoV**	**EV**	**229E**	**OC43**	**NL63**	**HKU1**	**MP**	**CP**	
HPIV-1	**58**	2	2	0	3	0	6	0	0	3	6	1	3	1	0	6	0	1.2
HPIV-2		**19**	2	0	1	0	6	0	1	0	1	2	3	0	0	2	0	0.4
HPIV-3			**99**	0	4	3	5	2	2	5	5	0	4	1	0	9	0	2.1
HPIV-4				**8**	0	0	1	1	0	0	1	0	0	0	0	1	0	0.2
infA					**833**	9	28	9	5	6	17	6	14	2	5	16	0	17.5
infB						**201**	4	0	5	1	5	0	0	0	0	4	0	4.2
RSV							**524**	11	7	8	44	6	3	5	1	13	1	11.0
ADV								**151**	3	4	10	4	4	4	1	5	0	3.2
HMPV									**164**	2	7	2	4	1	0	5	0	3.4
HBoV										**103**	7	0	3	1	3	10	0	2.2
EV											**226**	3	6	5	0	9	0	4.8
229E												**35**	6	2	0	1	0	0.7
OC43													**88**	2	0	6	0	1.9
NL63														**33**	0	1	1	0.7
HKU1															**20**	5	0	0.4
MP																**274**	0	5.8
CP																	**7**	0.1
1 pathogen	35	7	66	4	726	171	392	107	125	57	124	13	43	14	8	196	5	44.0
2 pathogens	18	8	23	4	92	29	104	31	33	32	80	15	30	13	9	65	2	12.4
≥3 pathogens	5	4	10	0	15	1	28	13	6	14	22	7	15	6	3	13	0	3.4

^**#**^HPIV: human parainfluenza virus, infA: influenza A virus, infB: influenza B virus, RSV: respiratory syncytial virus, ADV: adenovirus, HMPV: human metapneumovirus, HBoV: human bocavirus, EV: enterovirus, 229E: human coronavirus 229E, OC43: human coronavirus OC43, NL63: human coronavirus NL63, HKU1: human coronavirus HKU1, MP: *Mycoplasma pneumoniae*, and CP: *Chlamydophila pneumoniae.*

^*^Boldface indicates total numbers of pathogens detected. Note: multiple pathogens were isolated from some samples.

**Figure 1:**
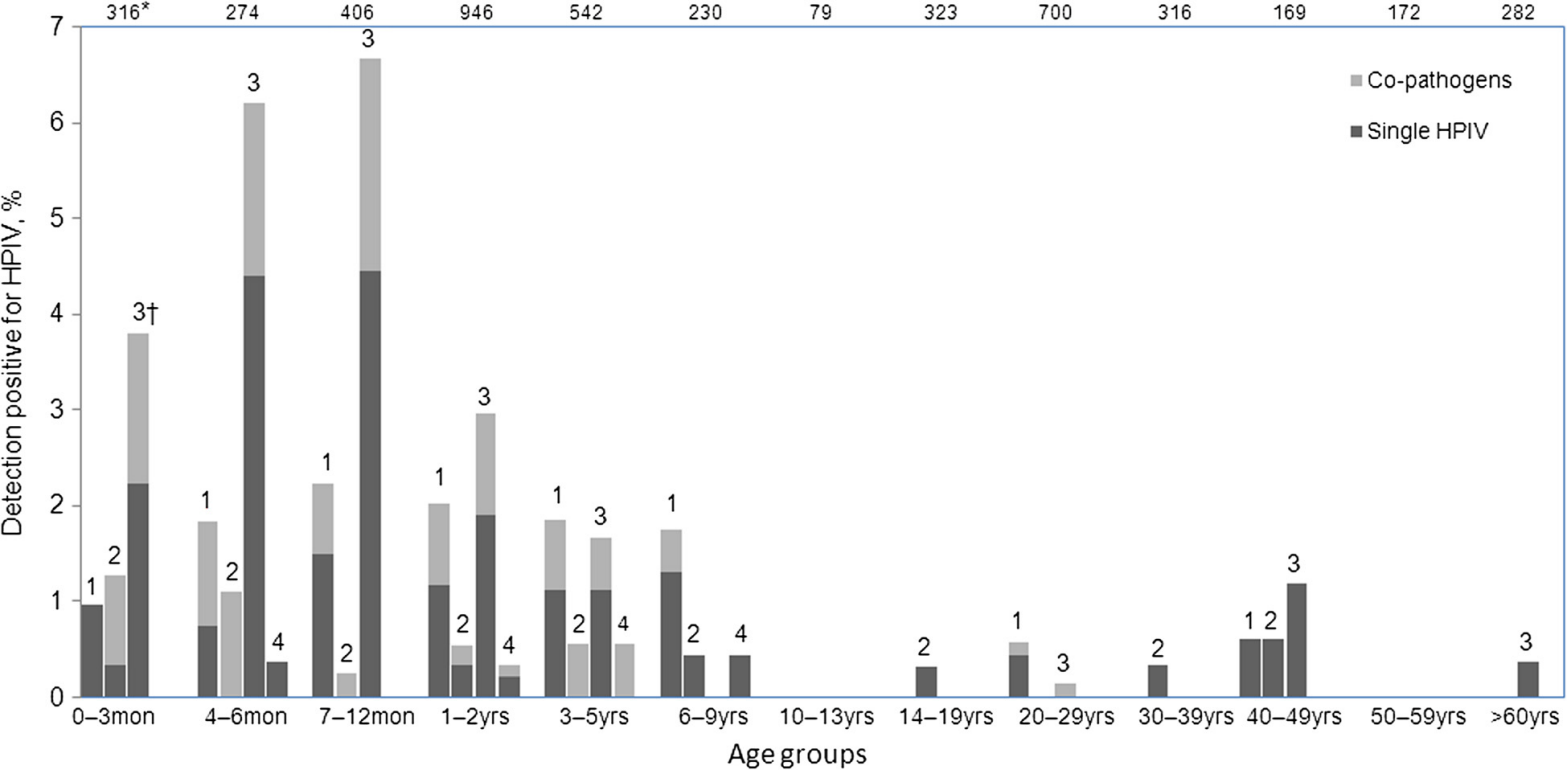
Distribution of HPIV-positive patients among different age groups.^*^Total number of patients of each age group. ^†^Type of HPIV.

HPIVs were identified in 178/4755 (3.7%) samples. Ninety-nine (2.1%) samples were positive for HPIV-3, 58 (1.2%) for HPIV-1, 19 (0.4%) for HPIV-2 and 8 (0.2%) for HPIV-4 (Table 1). Some samples were positive for multiple HPIV types, therefore the sum of these segments is more than 178. The age of patients positive for HPIV ranged from one month to 78 years. The male to female ratio was 139:39 in HPIV-positive patients and 2759:1818 in HPIV-negative patients (p<0.001). The child to adult ratio was 162:12 in HPIV-positive patients and 2627:1950 in HPIV-negative patients (p<0.001). The distribution and co-infection status of HPIV-positive patients are shown in Figure 1 according to different age groups. The large majority (160/178; 88.9%) of HPIV-positive samples were from paediatric patients younger than 5 years old (Figure 1), but no infant under one month of age was HPIV positive.

HPIV-3 was isolated from patients aged one month to 78 years. HPIV-3 was isolated predominantly from patients under 5 years of age, and the highest frequency was found in those aged 7–12 months (27/406; 6.7%). HPIV-1 was isolated from patients aged one month to 47 years, and HPIV-2 was isolated from patients aged 2 months to 41 years old. HPIV-1-positive patients were more evenly distributed across ages than HPIV-3-positive patients, but like HPIV-3 the highest frequency of HPIV-1 was detected from patients aged 7–12 months (9/406; 2.2%). HPIV-2 was not isolated as frequently as HPIV-3 or HPIV-1, but the highest frequencies of HPIV-2 were found in patients’ age groups of 0–3 months (4/316; 1.3%) and 4–6 months (3/274; 1.1%). Only 8 (0.2%) patients were HPIV-4 positive in this study, and patient age ranged from 5 months to 8 years.

66 of 178 (37.1%) HPIVs-positive-patients were co-infected with other 11/13 concerned pathogens except HCoV-HKU1 and CP. Pathogens with the highest frequency of co-infection with HPIV were RSV (18/66; 27.3%) and MP (18/66; 27.3%), followed by EV (13/66; 19.7%) and HCoV-OC43 (10/66; 15.2%) (Table 1). The vast majority of co-infections were in children (64/66; 97.0%), especially in patients under 5 years of age (63/66; 95.5%) (Figure 1). The co-infection rate of HPIV-2 was 63.2% (12/19), the rate of HPIV-4 was 50% (4/8) the rate of HPIV-1 was 39.7% (23/58) and the rate of HPIV-3 was 33.3% (33/99) (Table 1).

### Seasonal distribution of HPIVs

**Figure 2:**
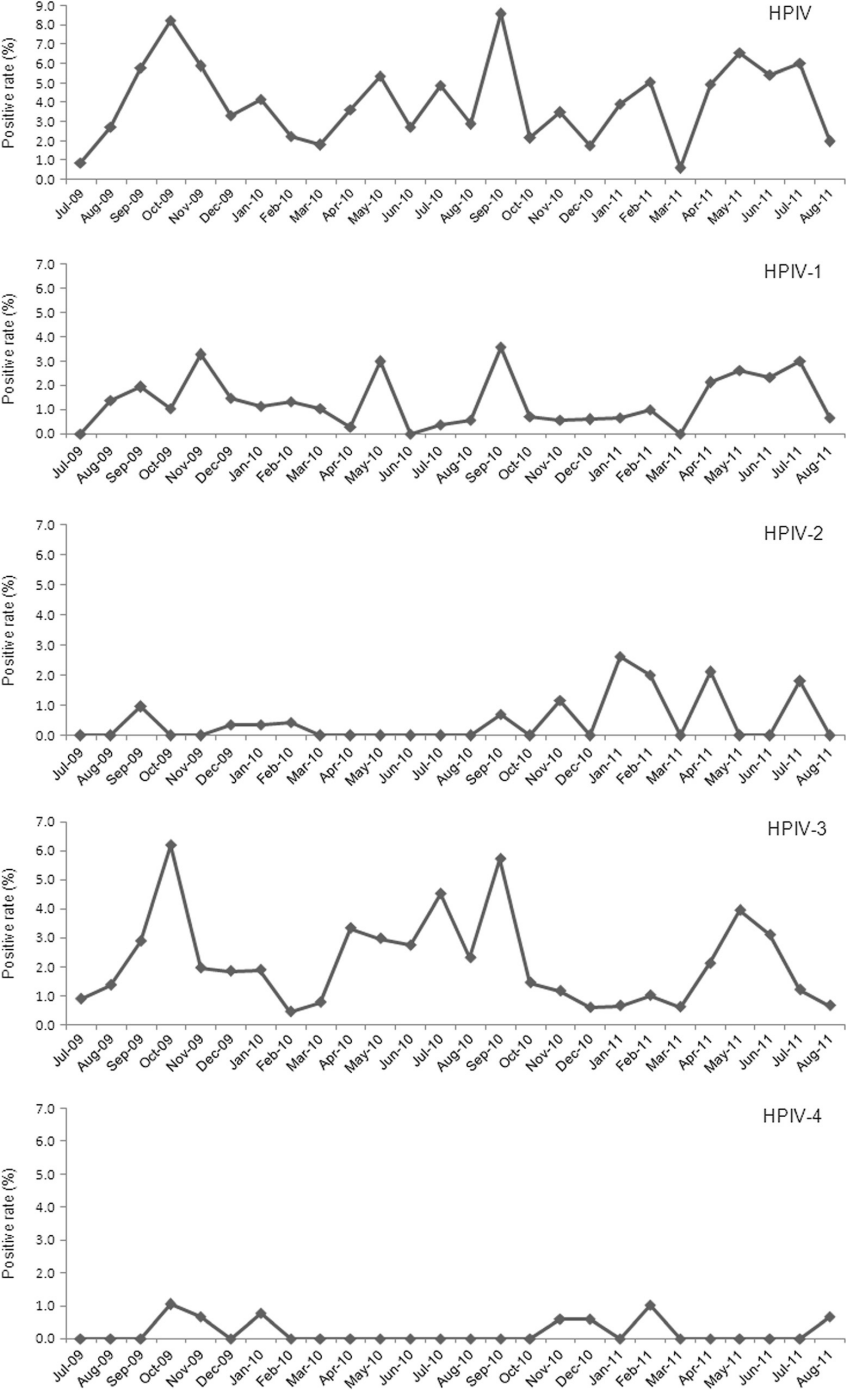
Seasonal distribution of the four HPIV types from July 2009 to August 2011.

In general, detection of HPIVs increased as autumn turned to winter (September 2009 to November 2009; September 2010 to October 2010) and summer turned to autumn (April 2011 to July 2011). HPIV-3 and HPIV-1, the two most frequently isolated types, predominantly drove these seasonal trends. HPIV-2 and HPIV-4 had different seasonal patterns, and were mainly isolated as winter turned to spring (December 2010 to March 2011) (Figure 2).

### Clinical characteristics of HPIV-positive patients

4447 of 4755 (93.5%) patients, and 174 of 178 (97.8%) HPIVs-positive patients’ clinical presentations were analyzed. Patients with incomplete clinical data available were excluded from the clinical data analysis. We compared the clinical characteristics of HPIV-positive to HPIV-negative patients, characteristics of each of the HPIV types, and characteristics of single HPIV infections to co-infections (Table 2).

**Table 2:** Clinical characteristics of participants

**Characteristics**	**HPIVs infection in 4447 patients**			**4 types distributions in 174 HPIVs-positive patients**					**Co-infected with HPIVs**		
	HPIV-positive (n=174)^*^	**HPIV-negative (n=4273)**	**p value**^**§**^	**HPIV-1 (n=57)**	**HPIV-2 (n=18)**	**HPIV-3 (n=97)**	**HPIV-4 (n=8)**	**p value**^**※**^	**Single HPIV (n=109)**	**Co-pathogens (n=65)**	**p value**^**#**^
Nasal obstruction	55(31.6)	1324(31.0)	0.862	20(35.1)	4(22.2)	31(32)	3(37.5)	0.767	34(31.2)	21(32.3)	0.878
Coryza	68(39.1)	1568(36.7)	0.523	22(38.6)	7(38.9)	44(45.4)	1(12.5)	0.306	47(43.1)	21(32.3)	0.157
Sneeze	10(5.7)	221(5.2)	0.738	1(1.8)	1(5.6)	7(7.2)	1(12.5)	0.421	7(6.4)	3(4.6)	0.964
Cough	164(94.3)	3426(80.2)	0.738	53(93)	17(94.4)	93(95.9)	7(87.5)	0.718	104(95.4)	60(92.3)	0.395
Expectoration	77(44.3)	1739(40.7)	0.35	11(19.3)	9(50)	49(50.5)	1(12.5)	**<0.001**	49(45)	28(43.1)	0.809
Pharyngeal discomfort^†^	33(19.0)	1428(33.4)	**<0.001**	16(28.1)	5(27.8)	12(12.4)	1(12.5)	0.073	22(20.2)	11(16.9)	0.596
Hoarseness	7(4.0)	68(1.6)	**0.015**	2(3.5)	1(5.6)	3(3.1)	1(12.5)	0.592	7(6.4)	0(0)	**0.037**
Abnormal pulmonary breathing sound^‡^	68(39.1)	1078(25.2)	**<0.001**	20(35.1)	7(38.9)	40(41.2)	4(50)	0.811	43(39.4)	25(38.5)	0.897
Dyspnoea	70(40.2)	1010(23.6)	**<0.001**	19(33.3)	7(38.9)	44(45.4)	3(37.5)	0.529	45(41.3)	25(38.5)	0.713
Increasing lung markings	10(5.7)	134(3.1)	0.056	4(7)	1(5.6)	4(4.1)	1(12.5)	0.718	6(5.5)	4(6.2)	0.859
Bronchopneumonia	24(13.8)	416(9.7)	0.079	11(19.3)	3(16.7)	10(10.3)	1(12.5)	0.464	17(15.6)	7(10.8)	0.372
Pneumonia	28(16.1)	428(10.0)	**0.01**	10(17.5)	3(16.7)	17(17.5)	0(0)	0.641	20(18.3)	8(12.3)	0.294
Fever (≥38°C)	97(55.7)	3004(70.3)	**<0.001**	33(57.9)	12(66.7)	53(54.6)	2(25)	0.532	57(52.3)	40(61.5)	0.235
Chill	6(3.4)	893(20.9)	**<0.001**	3(5.3)	0(0)	3(3.1)	0(0)	0.666	4(3.7)	2(3.1)	0.836
Dizziness	4(2.3)	543(12.7)	**<0.001**	3(5.3)	1(5.6)	0(0)	0(0)	0.125	3(2.8)	1(1.5)	0.605
Headache	7(4.0)	1063(24.9)	**<0.001**	4(7)	2(11.1)	1(1)	0(0)	0.092	6(5.5)	1(1.5)	0.198
Myalgia	3(1.7)	613(14.3)	**<0.001**	2(3.5)	1(5.6)	0(0)	0(0)	0.202	3(2.8)	1(1.5)	0.605
Debilitation	6(3.4)	1103(25.8)	**<0.001**	2(3.5)	1(5.6)	3(3.1)	0(0)	0.902	3(2.8)	3(4.6)	0.515
Vomiting	14(8.0)	326(7.6)	0.839	5(8.8)	2(11.1)	9(9.3)	0(0)	0.823	6(5.5)	8(12.3)	0.110
Poor appetite	27(15.5)	737(17.2)	0.553	11(19.3)	2(11.1)	13(13.4)	1(12.5)	0.736	17(15.6)	10(15.4)	0.970
Diarrhoea	16(9.2)	77(1.8)	**<0.001**	3(5.3)	1(5.6)	12(12.4)	0(0)	0.333	11(10.1)	5(7.7)	0.596
Convulsion	2(1.1)	63(1.5)	0.726	1(1.8)	0(0)	1(1)	0(0)	0.916	2(1.8)	0(0)	0.272
Rash etc.	5(2.9)	64(1.5)	0.15	5(8.8)	0(0)	5(5.2)	0(0)	0.443	4(3.7)	1(1.5)	0.416

^*^Data are No.(%) of each group. Percentages sum to over 100% because some patients had more than one diagnosis.

^†^Including pharyngeal dryness and pharyngalgia.

^‡^Including phlegm rale, wheeze rale, bubbling rale, moist rale, and rhonchi.

^§^Two-tailed χ^2^ test, testing the distribution of each illness or diagnosis between HPIV-positive and HPIV-negative patients.

^※^Two-tailed χ^2^ test, testing the distribution of each illness or diagnosis between the four HPIV types.

^#^Two-tailed χ^2^ test, testing the distribution of each illness or diagnosis between patients solely infected with HPIV and those co-infected.

Significant differences were seen between HPIV-positive and HPIV-negative patients for pharyngeal discomfort (p<0.001), hoarseness (p=0.015), “Abnormal pulmonary breathing sound” (p<0.001), “Dyspnoea” (p<0.001), “Pneumonia” (p=0.01), and “Diarrhoea” (p<0.001) (Table 2). In HPIV-positive samples, the proportion of patients with “Bronchopneumonia” was 13.8% (24/174) and the proportion with “Increasing lung marking” was 5.7% (10/174), although these proportions were not significantly different from HPIV-negative patients (p=0.079 and 0.056). HPIV-positive patients were less likely than HPIV-negative patients to present with each of the six symptoms of “Systemic influenza-like symptoms” (p<0.001) (Table 2).

The only significant difference in clinical characteristics between patients solely infected with HPIV and those co-infected was “Hoarseness” (p=0.037), present in 7 patients with “single HPIV” but no co-infected patient (Table 2).

The only significant difference in clinical presentation between HPIV types was “Expectoration,” in which 49/97 (50.5%) HPIV-3 positive patients, 9/18 (50%) HPIV-2 positive patients, 11/57 (19.3%) HPIV-1 positive patients and 1/8 (12.5%) HPIV-4 positive patients presented with the symptom (p<0.001) (Table 2).

Within HPIV-positive patients, 10/10 (100%) adult patients presented with systemic influenza-like symptoms, while 90/164 (54.9%) paediatric patients presented with these symptoms (p=0.005).

## Discussion

HPIVs are common respiratory pathogens and are important causes of URTI and LRTI [1,3,8,9]. Previous studies have predominantly focused on HPIV-1, HPIV-2 and HPIV-3 infection in children because of high positive rate and morbidity of three types of HPIV infection in children, therefore less is known about HPIV-4 infection and HPIV infection in adults [20]. In this study, we analysed the characteristics of the four HPIV types in children and adults with ARTI in Guangzhou, southern China over a 26 month period.

Of the pathogens investigated in this study, HPIVs were the sixth most frequently isolated. The predominant types were HPIV-3 and HPIV-1, which is consistent with previous reports [5-7]. HPIVs were isolated with higher frequency from males than females, similar to the previous study [21]. Immunity to HPIVs is incomplete, and infections occur throughout life [20]. In this study, HPIVs were detected in patients over a wide age distribution. However, many more children were infected than adults (p<0.001), and the vast majority of HPIV infections occurred in patients under 5 years of age. The four HPIV types differed in age distribution of patients infected. HPIV-3 was mainly detected in paediatric patients under 3 years old, while HPIV-1 and HPIV-2 were isolated from a broader age distribution than HPIV-3 (Figure 1). No infant under one month of age was HPIV positive. These results are in accordance with seroprevalence studies indicating that newborn infants have high levels of HPIV antibodies, and that these levels decrease substantially by 7 to 12 months of age [20]. In this study, HPIV-4 was only detected in children (4 months to 8 years old). This result is different from previous studies in which fairly equal infection rates were reported among infants younger than one year old, preschool children, school age children and adults [14-18]. The low HPIV-4 positivity in our study may have influenced our results.

Co-infection of HPIV with other respiratory pathogens was common for all four HPIV types, similar to previous reports [20-22]. RSV, MP, EV and HCoV-OC43 were the main co-detected pathogens in this study. Co-infections were mostly found in children, especially in patients under 5 years of age (Figure 1). This might indicate that immature immune systems of children leave them susceptive to potential pathogens. However, no significant differences in clinical presentation were seen between patients solely infected with HPIV and patients co-infected with HPIV and other respiratory pathogens (Table 2).

Biennial fall epidemics of HPIV-1 have been reported in previous studies [2,7,13,23]. HPIV-2 has been reported to cause infections biennially with HPIV-1, to alternate years with HPIV-1, or to cause yearly outbreaks [7,24,25]. HPIV-3 is reported to have occurred annually during April to June in the United States [2]. Only a small number of studies have studied the epidemiology of HPIV-4, and the numbers of infections were too low to clearly identify seasonal peaks in activity [2,14-18]. In this study, HPIVs were isolated throughout the year. Seasonal peaks of HPIVs, driven mostly by HPIV-3 and HPIV-1, occurred in the time when autumn turned to winter and summer turned to autumn (September 2009 to November 2009; September 2010 to October 2010; April 2011 to July 2011). These results differ from previous reports [2,7,13,23]. In addition, HPIV-3 and HPIV-1 did not show the competitive interaction described previously, where HPIV-3 activity appeared to be greater during years when HPIV-1 was not circulating [2]. The different geographic location might lead to the different seasonal distributions of HPIVs. HPIV-2 and HPIV-4 were not isolated as frequently as HPIV-3 and HPIV-1, and the seasonal peak of HPIV-2 and HPIV-4 was in the turn of winter to spring (December 2010 to March 2011). The frequency of detection of these two HPIV types increased when detection of HPIV-3 and HPIV-1 declined.

HPIVs can cause a spectrum of respiratory illness, and more “hoarseness” (p=0.015), “abnormal pulmonary breathing sound” (p<0.001), “dyspnoea” (p<0.001), and “pneumonia” (p=0.01) presented in HPIV-positive patients than HPIV-negative patients. In our study, more “diarrhoea” (p<0.001) presented in HPIV-positive patients than HPIV-negative patients suggesting pathogenic activity of HPIV in gastrointestinal illness (Table 2). Systemic influenza-like symptoms were not main presentations of HPIV-positive patients overall (Table 2), but HPIV-positive adult patients presented with significantly more systemic influenza-like symptoms than HPIV-positive paediatric patients (p=0.005). This result suggests that the clinical presentation of HPIV infection may differ by patient age as previously shown for HBoV [19].

The relationship between HPIV infection and neurologic disease has been studied for many decades. In a previous report, children hospitalized with HPIVs had serious febrile seizures [25]. In contrast, we found no significant difference in convulsion between HPIV-positive and HPIV-negative patients (p=0.726).

This study investigated presentations of the four HPIV types and attempted to distinguish between different types of HPIVs by clinical presentation. However, because all four HPIV types caused a wide spectrum of symptoms, distinguishing HPIV types by clinical characteristics alone was not possible as the previous study [26].

## Conclusions

HPIV infection led to a wide spectrum of symptoms, and LRTI was the significant presentation. Similar clinical manifestations were found in the patients with four different types of HPIVs. More diarrhoea found in HPIV-positive than HPIV-negative patients suggested pathogenic activity of HPIV in gastrointestinal illness. All 10 adults HPIV-positive patients suffered from systemic influenza-like symptoms suggested that the clinical presentation of HPIV infection may differ by patient age. Co-infections were common for the four HPIV types. However, no significant differences were seen in clinical presentation between patients solely infected with HPIV and patients co-infected with HPIV and other respiratory pathogens.

This study explored characteristics of the four HPIV types and provided novel insights into the epidemiology and clinical implications of HPIVs.

## Competing interests

The authors declare that they have no competing interests.

## Authors’ contributions

RZ, W-KL and QL designed the study. W-KL, QL, H-XL, X-KC, W-BH performed the pathogens testing. D-HC, Z-FY and SQ collected clinical data. All authors participated in the data analysis. W-KL, QL and RZ drafted the manuscript. All authors read and approved the final version of this manuscript.

## Pre-publication history

The pre-publication history for this paper can be accessed here:

http://www.biomedcentral.com/1471-2334/13/28/prepub
